# Supplementary figures and images for: Comparative Transcriptomic and Metabolomic Analyses of Differences in Trunk Spiral Grain in Pinus yunnanensis
Source: Int J Mol Sci. 2023 Sep 28;24(19):14658. doi: 10.3390/ijms241914658 (PMC10572851; doi:10.3390/ijms241914658)

a

Scores (OPLS-DA)

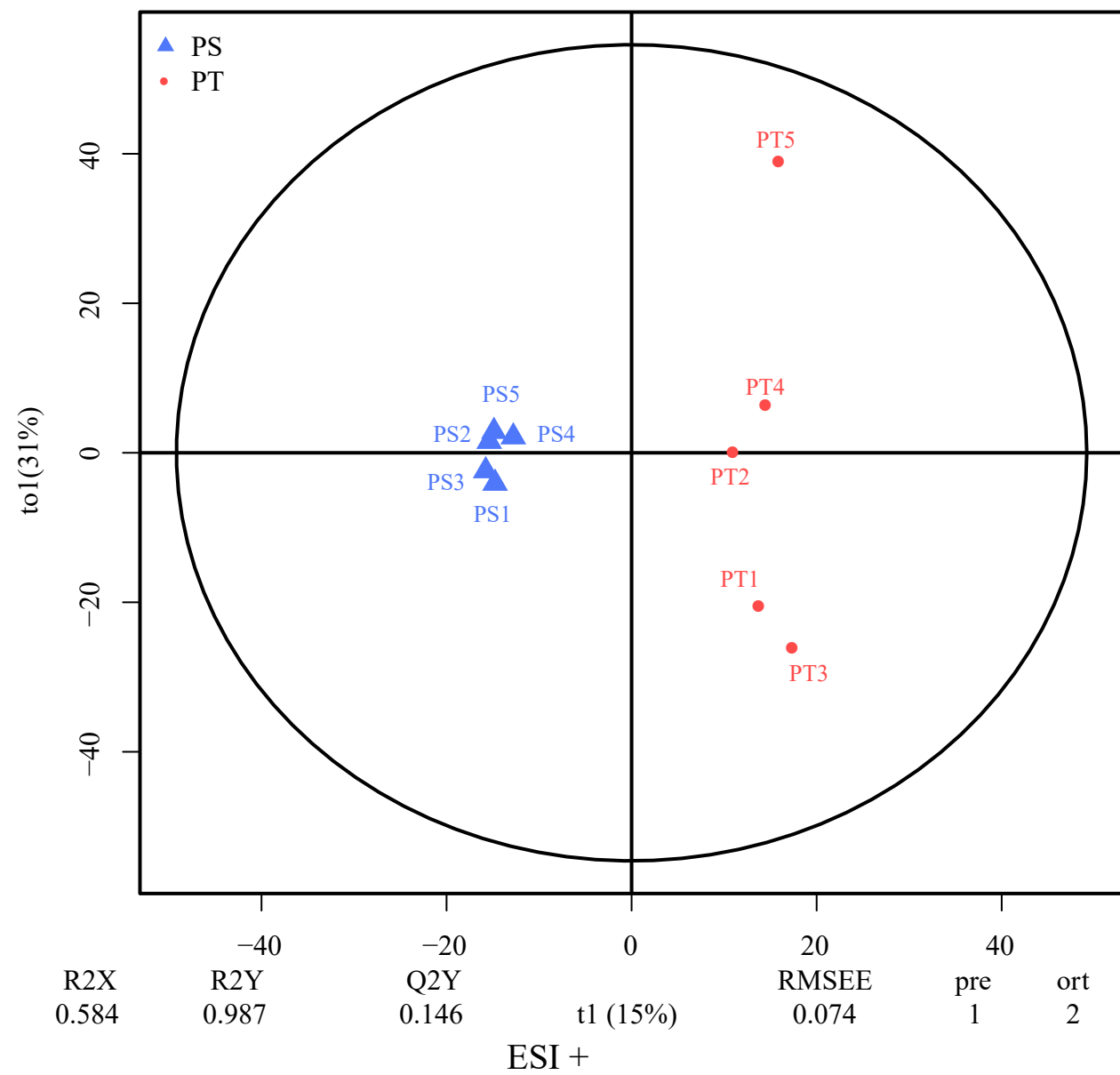

b

Scores (OPLS-DA)

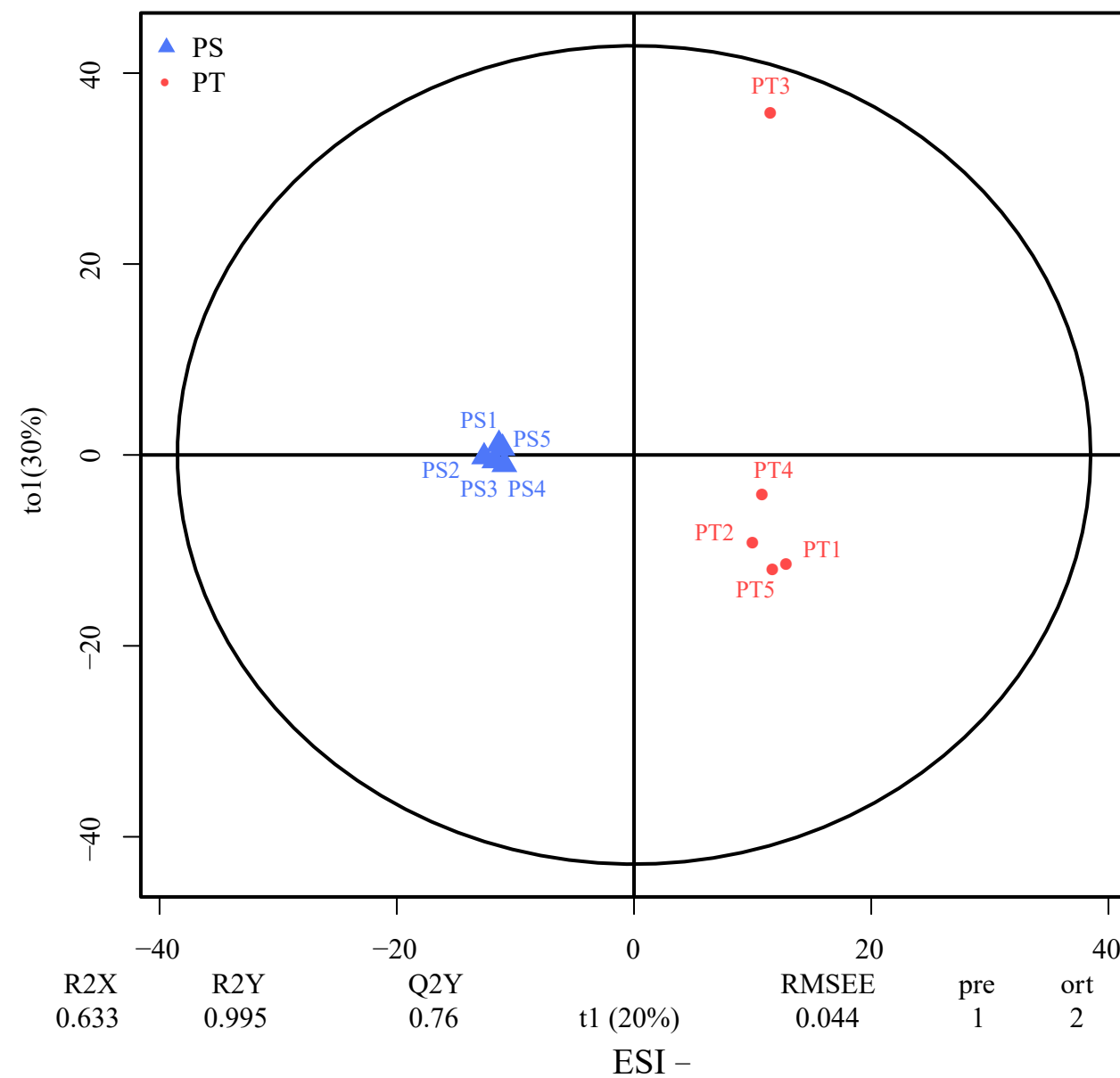

Supplement: Supplementary file 1 [file ijms-24-14658-s001.zip › Figure S1. OPDLA annalysis.pdf]

a

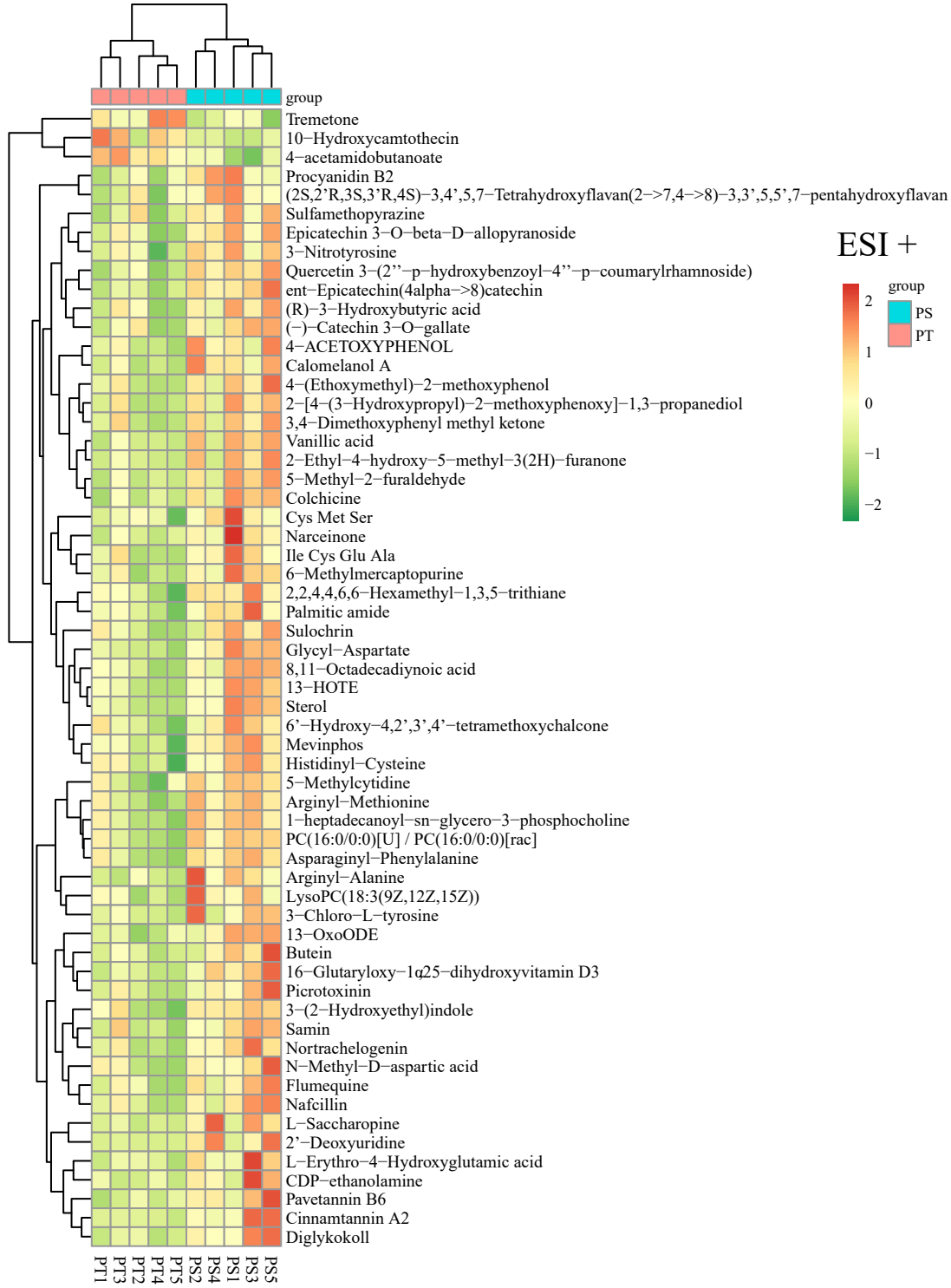

b

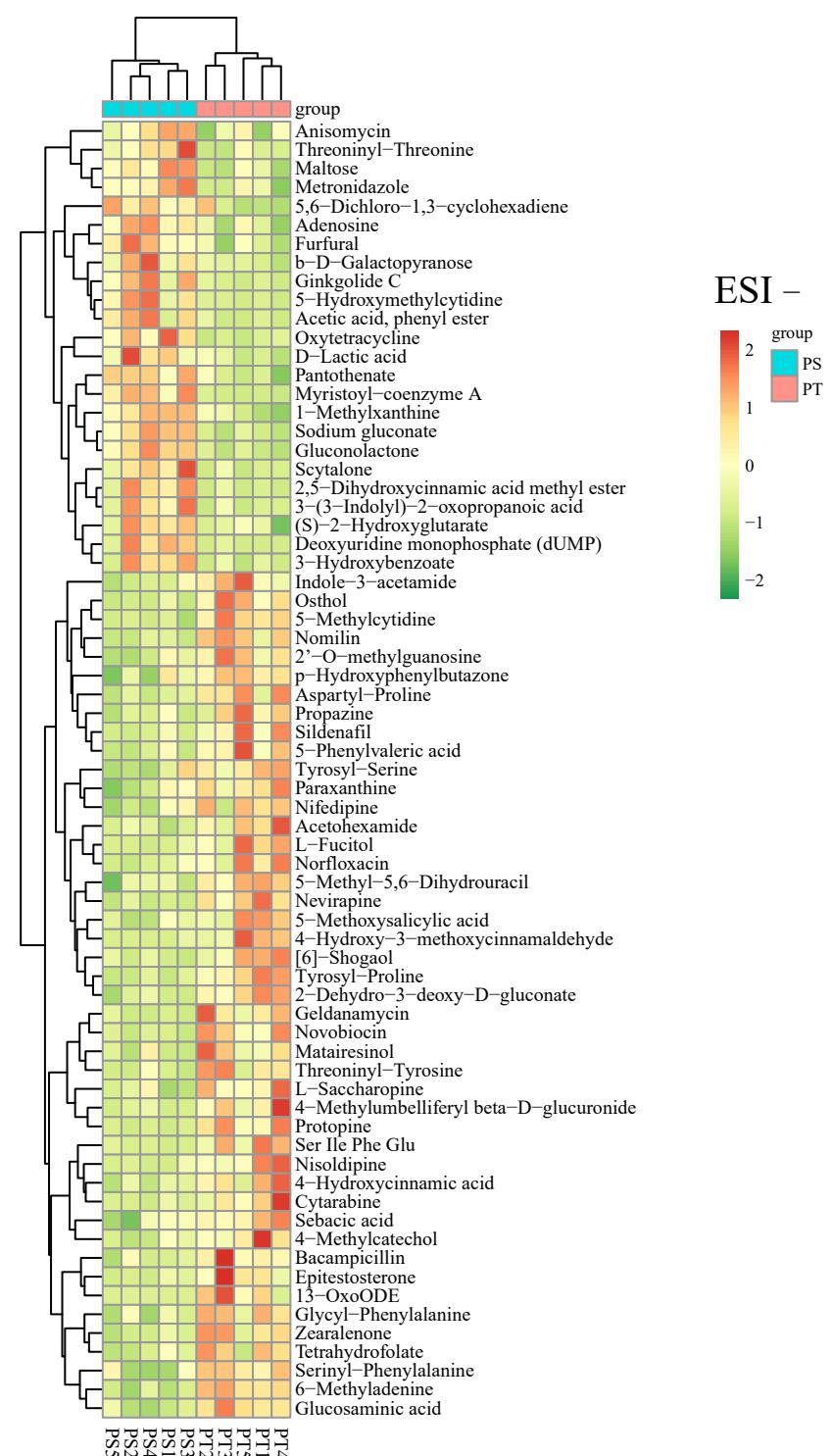

Supplement: Supplementary file 1 [file ijms-24-14658-s001.zip › Figure S2. Heatmap of DAMs.pdf]

a

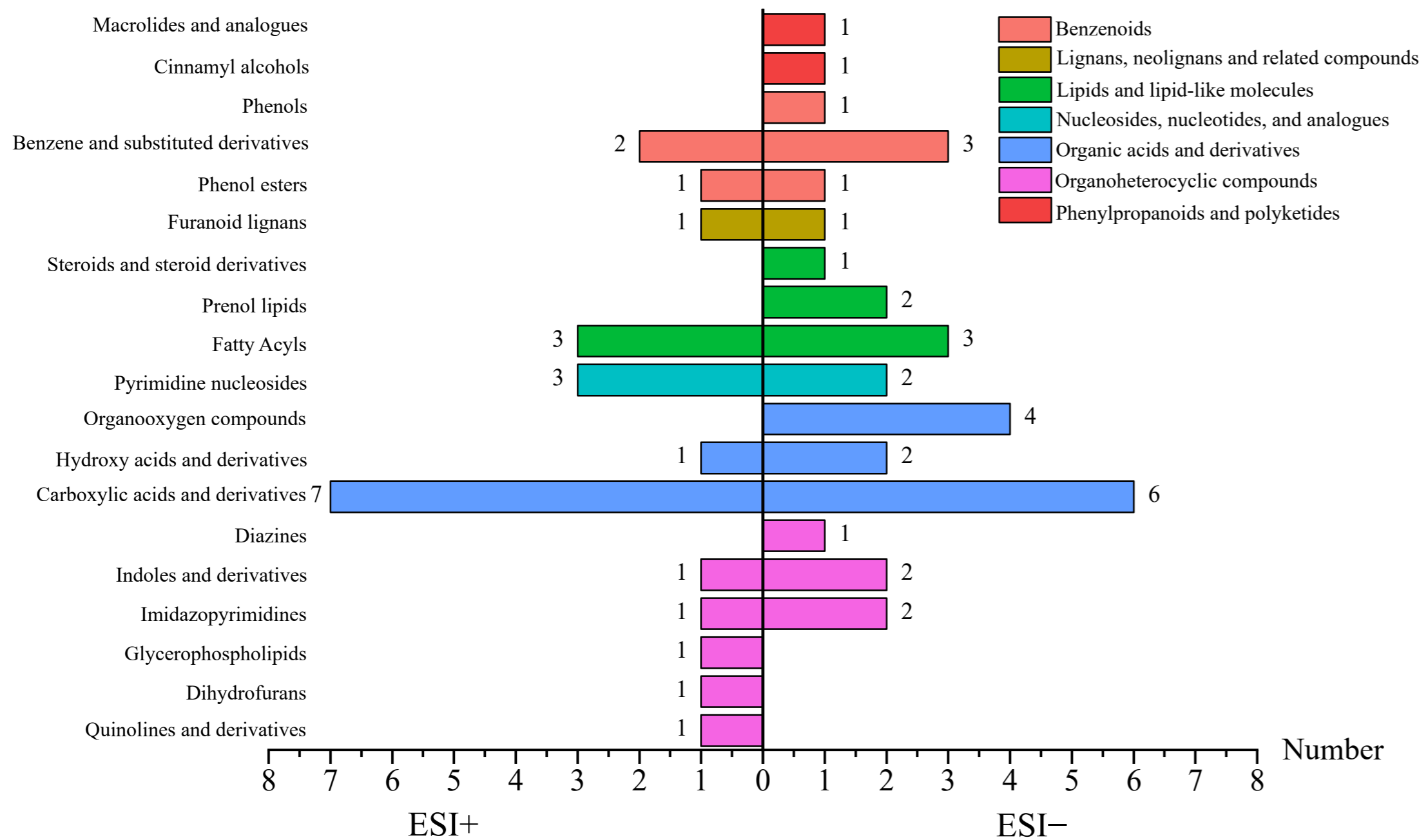

b

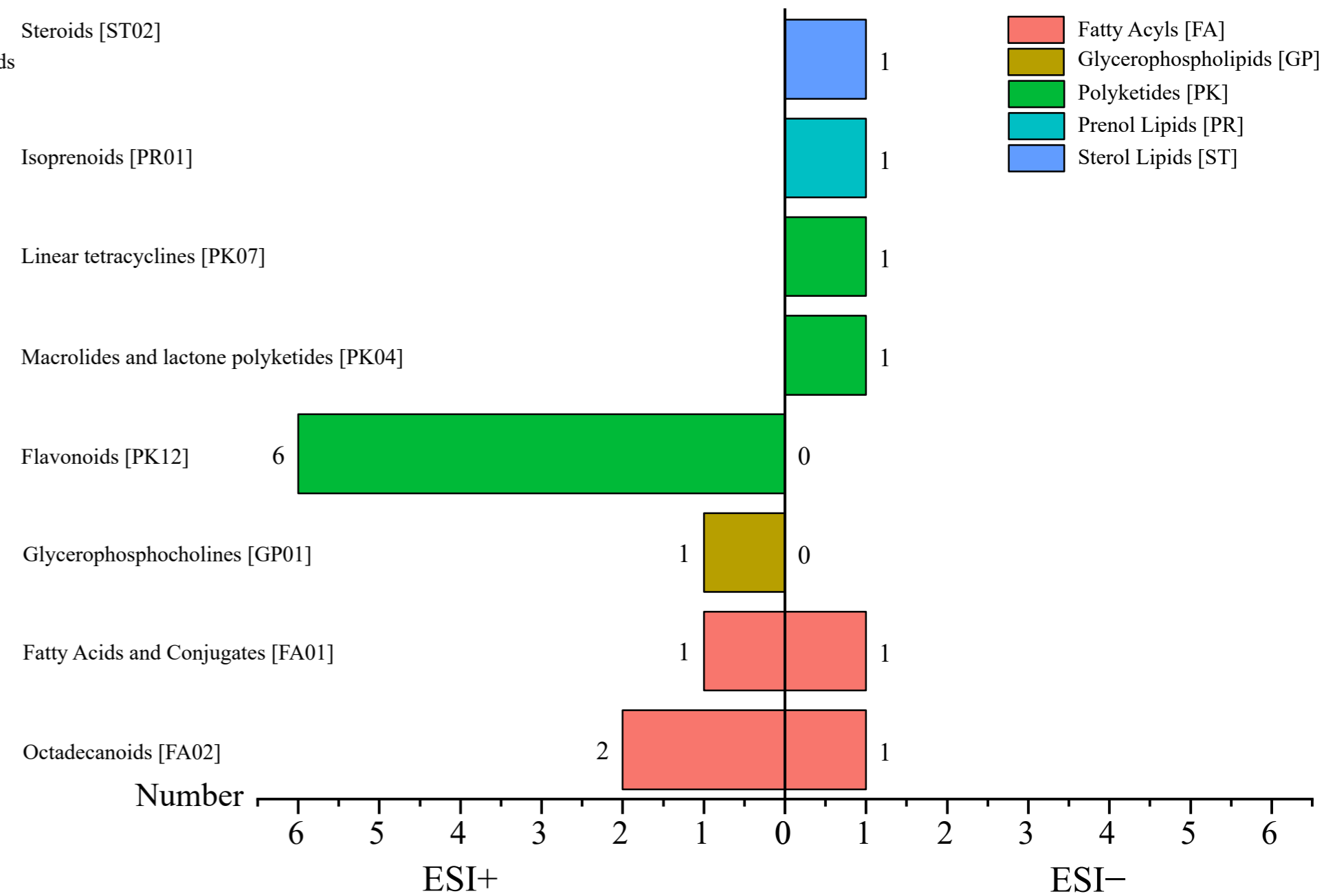

DAMs annotation in HMDB database

DAMs annotation in Lipidmaps database

Supplement: Supplementary file 1 [file ijms-24-14658-s001.zip › Figure S3. DAMs in HMDB and Lipid Map database.pdf]

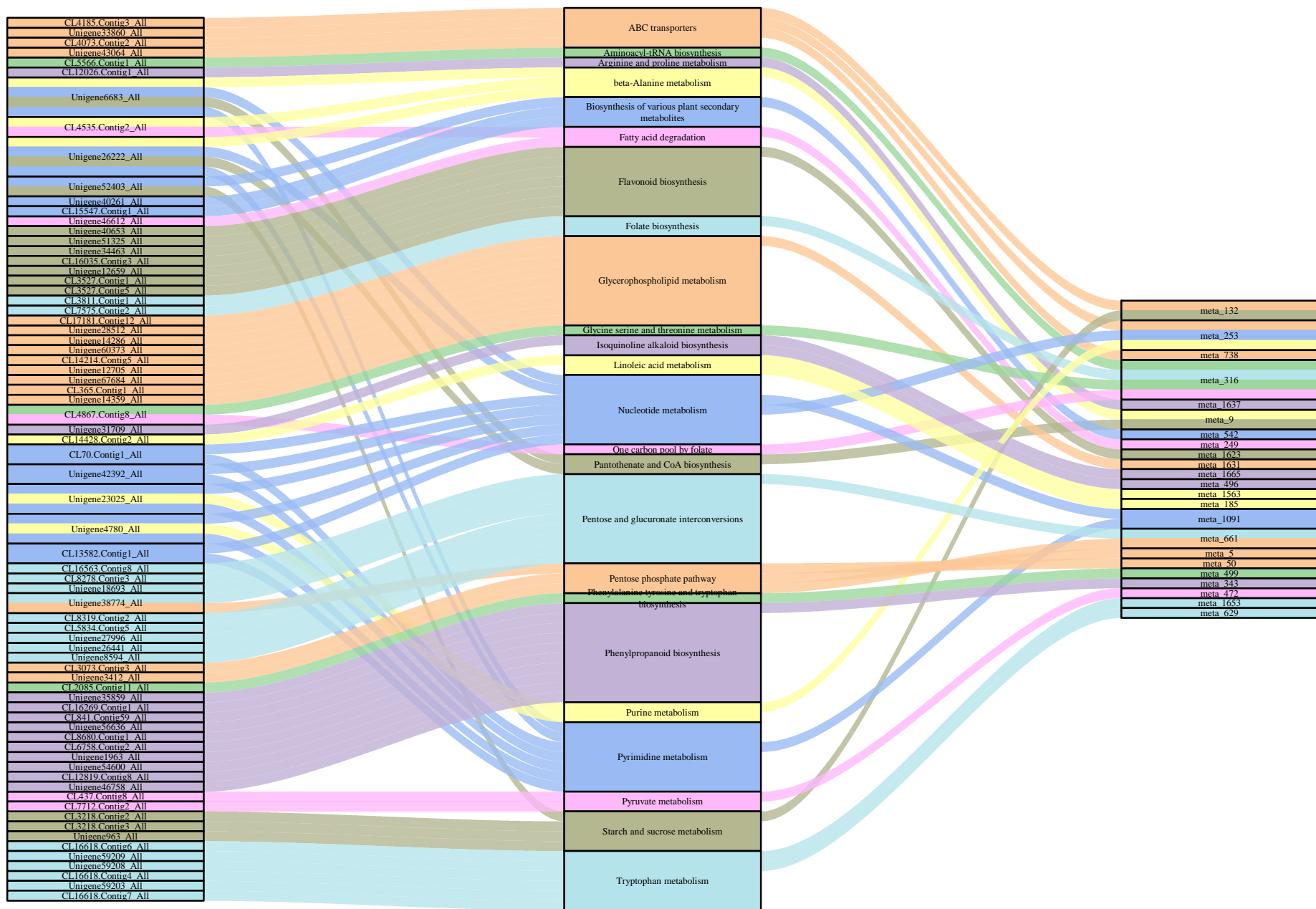

Transcript

Pathway

Metabolites

Supplement: Supplementary file 1 [file ijms-24-14658-s001.zip › Figure S4. Sankey of transcriptome and metabolome.pdf]

a

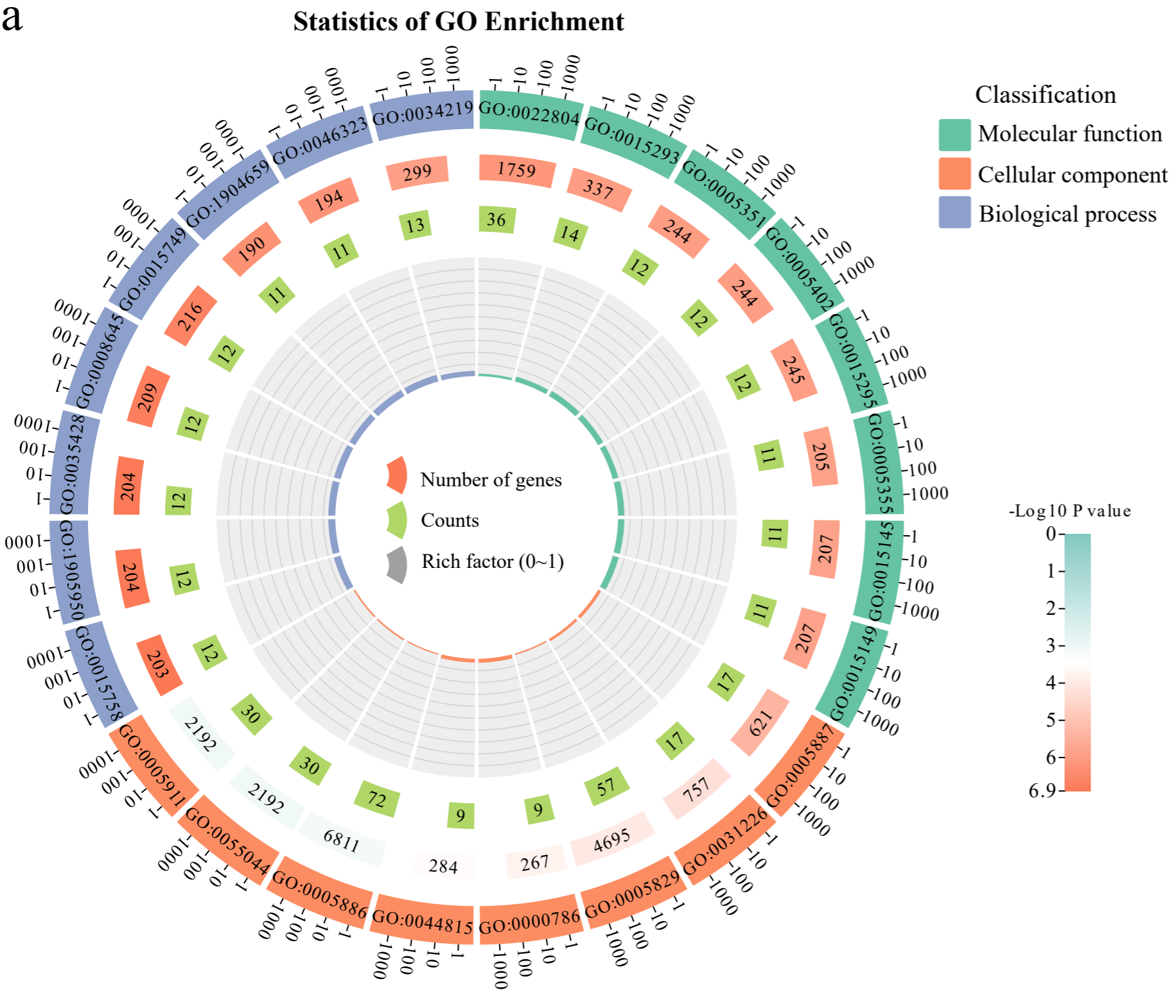

b

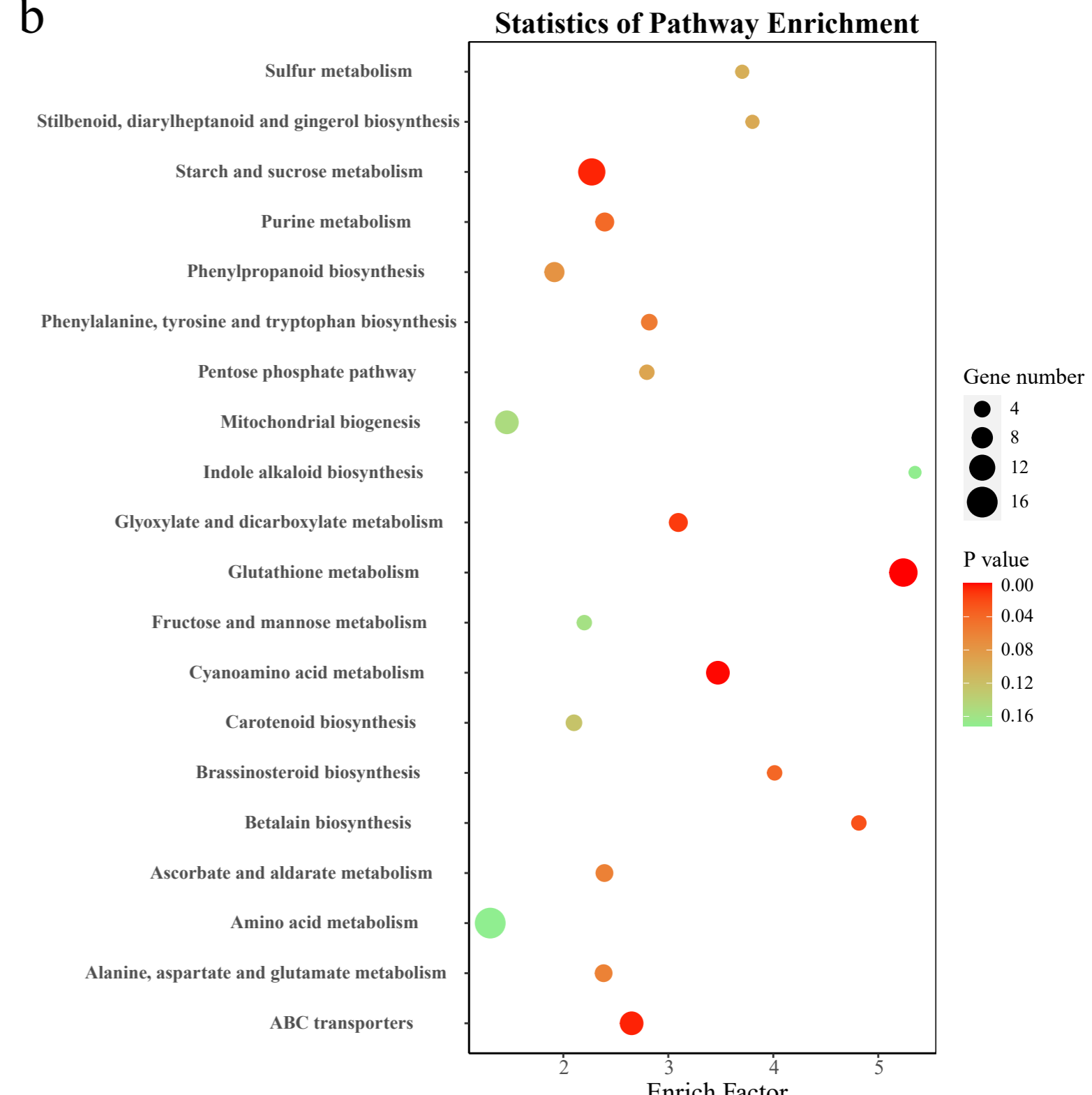

Supplement: Supplementary file 1 [file ijms-24-14658-s001.zip › Figure S5. GO and KEGG enrichment (a) GO enrichment,(b) KEGG enrichment in lightblue4 module.pdf]

Relative expression level

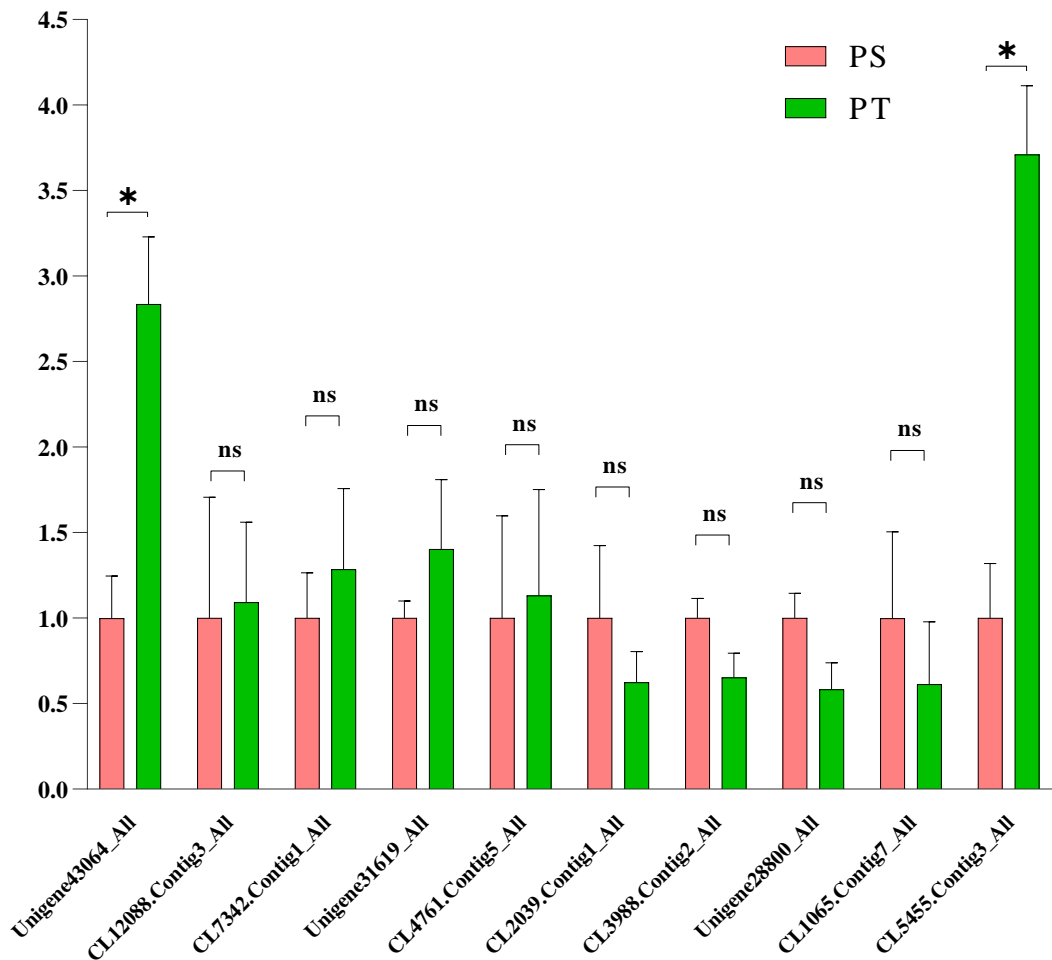

\* indicate significant differences at  $p < 0.05$ .

Supplement: Supplementary file 1 [file ijms-24-14658-s001.zip › Figure S6. RT-qPCR verification result.pdf]
